# The Effectiveness of School-Based Skills-Training Programs Reducing Performance or Social Anxiety: Two Randomized Controlled Trials

**DOI:** 10.1007/s10566-023-09736-x

**Published:** 2023-02-04

**Authors:** Amanda W. G. van Loon, Hanneke E. Creemers, Simone Vogelaar, Anne C. Miers, Nadira Saab, P. Michiel Westenberg, Jessica J. Asscher

**Affiliations:** 1 Child and Adolescent Studies, Utrecht University, Heidelberglaan 1, 3584 CS Utrecht, The Netherlands; 2 Forensic Child and Youth Care Sciences, University of Amsterdam, Nieuwe Achtergracht 127, 1018 WS Amsterdam, The Netherlands; 3 Developmental and Educational Psychology, Leiden University, Wassenaarseweg 52, 2333 AK Leiden, The Netherlands; 4 Graduate School of Teaching (ICLON), Leiden University, Kolffpad 1, 2333 BN Leiden, The Netherlands

**Keywords:** Adolescents, School-based interventions, Effectiveness, Stress, Mental health

## Abstract

**Background:**

Given that high levels of stress during adolescence are associated with negative consequences, it is important that adolescents with psychological needs are supported at an early stage, for instance with interventions at school. However, knowledge about the potential of school-based programs targeting adolescents with psychological needs, aimed at reducing school or social stress, is lacking.

**Objective:**

The current study aimed to investigate the effectiveness of two targeted school-based skills-training programs, addressing either skills to deal with performance anxiety or social skills.

**Methods:**

Two randomized controlled trials were performed with participants who self-selected to one of the programs. The sample comprised of *N* = 361 adolescents (*M*_age_ = 13.99 years, *SD* = 0.83) from various educational levels and ethnic identity backgrounds. The performance anxiety program included *N* = 196 participants (*N* = 95 in the experimental group), while the social skills program included *N* = 165 participants (*N* = 86 in the experimental group). MANCOVA’s were performed.

**Results:**

The performance anxiety program had a small effect on reducing adolescents’ test anxiety. Furthermore, for adolescents who attended more than half of the sessions, the program had small effects on reducing test anxiety and fear of failure. The program did not improve adolescents’ coping skills or mental health. The social skills program was not effective in improving social skills, social anxiety, and mental health.

**Conclusions:**

A relatively short, targeted program addressing skills to deal with performance anxiety can have the potential to reduce adolescents’ performance anxiety.

**Trial registration:**

International Clinical Trials Registry Platform (Netherlands Trial Register, number NTR7680). Registered 12 December 2018. Study protocol van Loon et al., (2019).

**Supplementary Information:**

The online version contains supplementary material available at 10.1007/s10566-023-09736-x.

## Introduction

Adolescence is a period of physical, psychological, and social developments (Christie & Viner, 2005), including puberty, the transition to secondary school, peer identification, and seeking independence. Moreover, adolescence is a period of elevated stress-sensitivity and increased risk of developing mental health problems (Andersen & Teicher, 2008; Romeo, 2013). High levels of stress throughout this developmental phase have been associated with various negative outcomes, including internalizing and externalizing problems (Snyder et al., 2017), burnout (Walburg, 2014), reduced well-being (Chappel et al., 2014), reduced academic performance (Arsenio & Loria, 2014), and school drop-out. To prevent adverse outcomes, it is important to support adolescents with psychological needs (i.e., vulnerable, at-risk adolescents) in a youth-friendly and stigma-free way (McGorry et al., 2013). Moreover, since early identification of and intervention for mental health issues may help prevent the development or reduce the severity and/or persistence of such disorders (McGorry et al., 2011), it is important to investigate the effects of early interventions for at-risk adolescents.

Interventions framed as stress reduction programs may be more appealing and less stigmatizing to adolescents with psychological needs than mental health interventions targeting for example anxiety or depression. Given that most adolescents experience stressors related to school or social situations at any given moment (Núñez-Regueiro & Núñez-Regueiro, 2021), and that they often cope with stress by talking about it (Camara et al., 2017), interventions focusing on stress reduction may be perceived by adolescents as a low-threshold and encouraging way to address their psychological needs. The school environment is particularly suitable for such interventions, as adolescents spend a large amount of their time at school (Eccles & Roeser, 2011), and the school context is important for adolescents’ emotional, social, and cognitive development (Roeser et al., 2000). Providing adolescents with appropriate tools to regulate their emotions and adequately cope with stress-inducing factors might prevent the development of mental health problems and promote adolescents’ well-being. The current study investigated the effectiveness of two school-based skills-training programs promoting adolescent mental health, by targeting either school or social stress.

A recent systematic review demonstrated that the most salient domains of negative stressors among adolescents are related to the family (e.g., issues with parents), the school (e.g., school pressure), the self (e.g., health issues), and peers (e.g., romantic issues; Núñez-Regueiro & Núñez-Regueiro, 2021). School-related stressors are often experienced by adolescents, and include school pressure (e.g., taking exams, workload), school performance (e.g., keeping up with school work), and academic difficulties (e.g., failure in exams, poor grades; Anniko et al., 2019; Núñez-Regueiro & Núñez-Regueiro, 2021). For instance, in the Netherlands, almost half of secondary students (aged 12–16 years) experience pressure from schoolwork, including homework and tasks or activities performed at school (47%; Boer et al., 2021), and one in three secondary students (aged 12–16 years) experience stress from school or homework (27%; Kleinjan et al., 2020). Furthermore, in various European countries, particularly higher income countries, increases in pressure from school and school stress have been demonstrated over the last two decades (Boer et al., 2021; Cosma et al., 2020, 2021; Stevens et al., 2018). School-based distress is positively associated with performance anxiety (Fernández-Sogorb et al., 2021), where individuals experience fear of failure, the fear to be unable to meet certain expectations of themselves or others, or test anxiety (i.e., a situation-specific form of (performance) anxiety evoked by evaluative or testing situations; von der Embse et al., 2017). Hence, improving skills to deal with performance anxiety may be a promising target to reduce school-related stress and prevent mental health problems in adolescents.

Previous literature demonstrated the potential of school-based interventions targeting test anxiety. Systematic reviews and recent studies showed that school-based interventions consisting of biofeedback and cognitive and/or behavioral frameworks had small to large effects on reducing adolescents’ test anxiety (O’Driscoll & McAleese, 2021; Putwain & von der Embse, 2021; Soares & Woods, 2020; von der Embse et al., 2013). In addition, either directly or indirectly through a reduction in test anxiety, school-based test anxiety prevention and intervention programs also improved mental health outcomes of adolescents, including reduced physiological stress (Bradley et al., 2010) and internalizing problems (O’Driscoll & McAleese, 2021; Weems et al., 2014), and resulted in an increase in self-esteem (Yahav & Cohen, 2008) and self-compassion (O’Driscoll & McAleese, 2021). Nevertheless, knowledge on the effectiveness of school-based interventions targeting test anxiety remains limited for a number of reasons. First, systematic reviews indicated that not all interventions were effective in reducing test anxiety (Soares & Woods, 2020; von der Embse et al., 2013), suggesting differences in effectiveness (e.g., between interventions, samples, or studies) that are not yet understood. Second, methodological issues limit the robustness of previous studies’ results. More specifically, only half of the studies used a randomized controlled design, which provides the strongest evidence for causal relations between intervention and outcome. Third, half of the studies focused on classroom or universal interventions rather than targeted interventions (i.e., aimed at at-risk selective samples), leaving the effectiveness for at-risk students and targeted interventions partly unknown. Fourth, previous studies mainly focused on interventions to reduce test anxiety, while research on interventions targeting the broader performance anxiety in adolescents is scarce and mainly focuses on specific populations, such as music students (Burin & Osório, 2016), athletes (Cadieux et al., 2021), or young children (Blanco et al., 2015). Although results from these studies are encouraging, they cannot be generalized to the larger group of adolescents. Overall, even though available research in adolescents yields promising results regarding the potential of school-based interventions to reduce test and performance anxiety, more robust research on the effectiveness of targeted school-based interventions addressing skills to deal with performance anxiety in adolescents is necessary.

Besides school-related stressors, stressors related to social situations are also common and intense among adolescents, including issues with parents (e.g., conflicts, arguments, misunderstandings), romantic relationships, and peer pressure (Anniko et al., 2019; Núñez-Regueiro & Núñez-Regueiro, 2021). For instance, various studies suggest that a substantial proportion of adolescents in European countries experience limited family and peer support (28% and 40%, respectively; Inchley et al., 2020; Stevens et al., 2018), and that one in 10 Dutch adolescents experience stress related to social problems (e.g., problems or disagreements at home, quarrels with others; Kleinjan et al., 2020). As dysfunctional relationships and factors that disrupt the relationship or interaction with others, such as isolation, rejection, or disagreements can evoke stress (Ditzen & Heinrichs, 2014; Juth & Dickerson, 2013), improving social skills might reduce social stress and promote mental health in adolescents. More specifically, learning or improving skills to initiate and maintain positive social relationships (e.g., assertiveness, communication skills) may protect against mental health problems among adolescents (Eskin, 2003). Indeed, previous research demonstrated that assertiveness training reduced levels of stress and improved psychological well-being and self-esteem in adolescents (Parray & Kumar, 2017).

A meta-analysis and various studies have demonstrated the potential of school-based programs to improve social and emotional skills, as well as mental health outcomes (e.g., emotional distress, internalizing problems, self-concept) and behavioral problems (Durlak et al., 2011; Gaspar et al., 2018; Schonert-Reichl & Lawlor, 2010). Yet, only universal interventions (i.e., aimed at the entire student body) were examined (Durlak et al., 2011; Gaspar et al., 2018; Schonert-Reichl & Lawlor, 2010), while previous research demonstrated that targeted programs (i.e., aimed at at-risk selected samples) are more effective than universal interventions (Feiss et al., 2019; van Loon et al., 2020; Werner-Seidler et al., 2017). Overall, more research on the effectiveness of targeted school-based interventions addressing social skills in adolescents is needed.

Taken together, although previous research suggests that universal school-based intervention programs have the potential to reduce test anxiety and improve social skills, as well as improve mental health outcomes, more knowledge about the potential of targeted school-based skills-training programs addressing either skills to deal with performance anxiety or social skills is needed to strengthen their evidence base. Therefore, the current study aimed to examine the effectiveness of two targeted school-based skills-training programs promoting adolescent mental health by targeting either school or social stress, where participants self-selected to either the performance anxiety program or the social skills program (van Loon et al., 2019). With two randomized controlled trials, we examined the effectiveness of (1) the performance anxiety program in improving skills to deal with performance anxiety (i.e., program targets), reducing performance anxiety (i.e., direct program outcomes), and improving mental health (i.e., reduced stress, internalizing and externalizing problems, and increased well-being and self-esteem); and (2) the social skills program in improving social skills (i.e., program targets), reducing social anxiety (i.e., direct program outcome), and improving mental health (i.e., reduced stress, internalizing, and externalizing problems, and increased well-being and self-esteem). We expected that the performance anxiety program would improve adolescents’ coping skills, reduce performance anxiety (i.e., fear of failure and test anxiety) and improve mental health (i.e., reduce stress, internalizing and externalizing problems, and increase well-being and self-esteem). We expected that the social skills program would improve adolescents’ social skills, reduce social anxiety and improve mental health.

## Method

### Design and Procedure

This study was performed in the context of a larger project, that aimed to strengthen the connection between secondary education and youth care by offering preventive interventions in schools. The goal was to identify vulnerable, at-risk adolescents and provide them with appropriate help, following a Response to Intervention model (RtI): a three-tiered approach with universal, targeted, and intensive interventions (Kearney & Graczyk, 2014). First, classes of students received psychoeducation about stress (i.e., Stress Lessons; tier 1 of the RtI model: universal intervention for all students). The goal of the Stress Lessons was to teach adolescents about stress, that is, how a body reacts to stress, how to recognize stress, and how to prevent and cope with stress. As previous studies demonstrated that psychoeducation programs reduced stigma of mental health problems and encouraged help-seeking in educational settings (Han & Chen, 2014; Waqas et al., 2020), the Stress Lessons served to reduce stigma about signing-up to a follow-up (mental health) intervention. Next, after these three educative lessons, students were offered the possibility to self-register for a skills-training program at their school to learn more about dealing with school or social stress (i.e., tier 2 of the RtI model: targeted intervention for a selected sample of students). Students could choose between the performance anxiety program or the social skills training, based on their own needs (e.g., related to either school or social stress). Students and parents received information about the performance anxiety training (i.e., “with this group training your child learns to deal with negative thoughts, practices relaxing and learns to deal with pressure and stress at school”) and the social skills training (i.e., “with this group training your child learns how to stand up for him/herself, to give his/her own opinion and learns to deal better with his/her emotions, feelings, and behavior”). All students who self-registered enrolled in one of the skills-training programs (students were not screened). These students were asked to participate in the present study. Tier 3 of the RtI model contains intensive interventions directed at students with severe and/or complex problems (i.e., requiring an individualized approach), which was outside the scope of the current study.

Two randomized controlled trials were conducted for both skills-training programs. Students (and parents) received written information about the skills-training programs that were offered and received an information letter about the corresponding study (on paper and via email). Effort was put into promoting the skills-training programs to students (and parents), including motivating mentors and school staff, giving presentations (about the programs and study) during classes, sending a promotion video (by trainers of the programs), and telephoning parents. Students and parents provided active informed consent for the students’ participation in the study. Participants were randomly allocated by the first author (stratified for educational level, using a computerized randomization in a 1:1 ratio) into the experimental (for which the training started immediately) or the waitlist control group. Before the start of the skills-training programs (T1) and immediately after completion of the intervention programs (T2), program targets (i.e., skills to deal with performance anxiety or social skills), direct program outcomes (i.e., performance or social anxiety), and mental health outcomes (i.e., stress, internalizing and externalizing problems, self-esteem, and well-being) were assessed in both groups. Participants filled in the questionnaires individually in small groups (i.e., not during classes), under supervision of one or multiple researchers. Filling out the questionnaires took approximately 45 min. The current study is registered in the International Clinical Trials Registry Platform (Netherlands Trial Register; number NTR7680) and the Ethical Committee Psychology of Leiden University approved the design of the study (number CEP19-1210/577) (for study protocol, see van Loon et al.,^ 2019^). The authors have no conflict of interest.

### Participants

This project was performed at nine secondary schools located in one of the four largest cities in the Netherlands. Dutch secondary school starts around the age of 12, and contains four different tracks: a track preparing students for work (5-year track; practical education), a track preparing students for vocational training (4-year track; preparatory vocational education), a track preparing students to study at universities of applied sciences (5-year track; senior general education) and a track preparing students for university education (6-year track; preparatory university education). As it is essential that various groups are represented in study samples, especially socially disadvantaged groups (e.g., groups of lower socioeconomic status) (Bonevski et al., 2014), the programs were evaluated in a heterogeneous sample of adolescents, with different ethnic identity backgrounds and educational levels. Based on a power of 0.80, an alpha of 0.05, and a medium effect size of 0.25 we aimed to include at least *N* = 130 participants (*N* = 65 for the experimental and *N* = 65 for the waitlist control group) for each school-based skills-training program to ensure there was enough power for the analyses (van Loon et al., 2019).

**Fig. 1:**
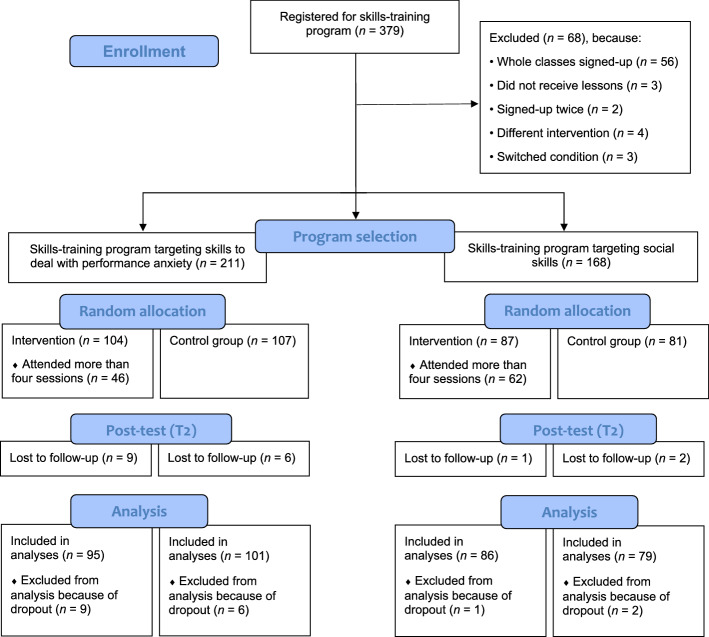
Flow chart of both school-based skills-training programs

In total, 379 participants registered for the skills-training programs (see Fig. 1 for the flow chart of the study). More than half of the participants registered for the performance anxiety program (55.7%; *N* = 211, with *n* = 104 in the experimental group), while the other participants registered for the social skills program (44.3%; *N* = 168, with *n* = 87 in the experimental group). Some participants were excluded from the sample because they were not targeted as intended (*N* = 68)^1^ (Fig. 1). From the total sample, 18 participants (4.7%, with *n* = 15 in performance anxiety program and *n* = 3 in the social skills program) were lost to follow-up (i.e., dropped out of the study at T2). For the performance anxiety program, dropouts were more often participants from the lowest educational level at T1 (χ^2^ = 6.831, *p* = 0.029). For the social skills program, dropouts had higher levels of well-being at T1 (*t* = − 5.905, *p* = 0.002). There were no other differences at T1 between participants and dropouts regarding demographics (i.e., age, number of siblings, gender, educational level, school year, financial problems, country of birth, ethnic identity, and living situation) and study variables. Dropouts were excluded from the analyses (see Fig. 1). The final sample comprised *N* = 361 adolescents between 12 and 17 years old (*M*_age_ = 13.99 years, *SD* = 0.83, 51.6% female). Table 1 presents further details about demographics of the samples. This study followed the Consolidated Standards of Reporting Trials guidelines (CONSORT; Begg et al., 1996), as outlined in Fig. 1.

**Table 1:** Demographics at baseline (T1) and differences at T1 between the experimental and control group for both school-based skills-training programs

Variables	Performance anxiety program^a^			Social skills program^b^		
	Experimental group (*N* = 95)	Control group (*N* = 101)	Differences at T1	Experimental group (*N* = 86)	Control group (*N* = 79)	Differences at T1
	*M* (*SD*)	*M* (*SD*)	*t* (*p*)	*M* (*SD*)	*M* (*SD*)	*t* (*p*)
Age	14.14 (0.75)	14.11 (0.82)	0.262 (.794)	13.86 (0.89)	13.81 (0.84)	0.382 (.703)
Number of siblings	1.74 (1.24)	1.94 (1.30)	− 1.120 (.264)	1.95 (1.48)	1.94 (1.31)	0.077 (.939)
	*N* (%)	*N* (%)	χ^*2*^ (*p*)	*N* (%)	*N* (%)	χ^*2*^ (*p*)
Female	58 (61.1)	45 (44.6)	5.344 (.015)	41 (47.7)	42 (53.2)	0.496 (.292)
Educational level			0.119 (.950)			0.022 (1.000)
Practical-prevocational education	31 (32.6)	34 (33.7)		38 (44.2)	35 (44.3)	
Prevocational/senior general education	26 (27.4)	29 (28.7)		20 (23.3)	19 (24.1)	
Senior general-preuniversity education	38 (40.0)	38 (37.6)		28 (32.6)	25 (31.6)	
School year			1.929 (.593)			3.668 (.303)
First year	12 (12.6)	17 (16.8)		18 (20.9)	24 (30.4)	
Second year	62 (65.3)	62 (61.4)		52 (60.5)	41 (51.9)	
Third year	14 (14.7)	11 (10.9)		9 (10.5)	11 (13.9)	
Fourth year	7 (7.4)	11 (10.9)		7 (8.1)	3 (3.8)	
Financial problems	5 (5.3)	6 (5.9)	0.042 (.542)	5 (5.8)	3 (3.8)	0.363 (.408)
Country of birth: the Netherlands	78 (82.1)	87 (86.1)	0.598 (.282)	78 (90.7)	70 (88.6)	0.195 (.426)
Ethnic identity			1.124 (.601)			1.809 (.411)
Western	50 (52.6)	59 (58.4)		46 (53.5)	36 (45.6)	
Western-non-Western	12 (12.6)	14 (13.9)		13 (15.1)	18 (22.8)	
Non-Western	33 (34.7)	28 (27.7)		27 (31.4)	25 (31.6)	
Living situation: living with both parents	67 (70.5)	63 (62.4)	1.456 (.146)	59 (68.6)	55 (69.6)	0.020 (.511)

^a^Skills-training program targeting skills to deal with performance anxiety; ^b^skills-training program targeting social skills

Our sample contained three cohorts that were differently affected by the COVID-19 pandemic. The first cohort of data collection started and finished in the school year 2018/2019 at three schools (*N* = 89 participants, see Supplementary Table 1). The second cohort started in the school year 2019/2020 at nine (six additional) schools. Two schools finished the skills-training programs before the COVID-19 pandemic and school closings (*N* = 55 participants). Due to the COVID-19 pandemic and the abrupt closing of the schools (from March 16, 2020), the skills-training programs that had started in February 2020 at five schools were cancelled after 2–4 sessions. At four of these schools, the programs restarted in September 2020 when the schools reopened (i.e., T1 assessments were repeated for these participants). Furthermore, two schools had not yet started with the skills-training programs in February 2020. Programs at these schools were postponed to September 2020 (total of *N* = 217 participants). Unfortunately, having to restart or postpone the programs in these six schools resulted in drop-out from the intervention (not the assessments): half of the participants at these schools did not attend any session of the performance anxiety program (*N* = 30 of 62 participants) and a quarter did not attend any session of the social skills program (*N* = 11 of 48 participants).

### School-Based Skills-Training Programs

The skills-training programs were provided at the schools by trained professionals from three youth care organizations (*N* = 23 trainers, 57% women, *M*_*age*_ = 34 years). All trainers had at least higher vocational education (i.e., senior general education) and had at least one year experience as a trainer (*M* = 5 years). The programs were adjusted from existing performance anxiety and social skills programs offered by the participating youth care organizations. Program duration was shortened to fit the schedule of schools. Each skills-training program consisted of seven 45-min small-group sessions during consecutive weeks. The groups consisted of approximately eight students (range of 3–16 participants), with one or two professional trainers per group.

#### Performance Anxiety Program

The performance anxiety program consisted of psychoeducation (e.g., different forms of performance anxiety, consequences of stress), cognitive coping strategies (e.g., negative thought restructuring, managing emotions), dealing with (school) pressure (e.g., keeping focus, planning skills), and relaxation techniques (e.g., breathing exercise). With regard to program integrity, on average, 90% of the program assignments were correctly implemented (i.e., assignments were executed consistent with the protocol, self-reported by the trainers after each session). In the remainder, program assignments could not be completed within the time frame.

#### Social Skills Program

The program targeting social skills consisted of social skill building (e.g., listening to others, identifying personal qualities, giving own opinions, standing up for yourself, setting boundaries) and cognitive coping strategies (e.g., managing emotions). Regarding program integrity, on average, 89% of the program assignments were correctly implemented. Again, the other assignments could not be completed due to time constraints.

### Control Group

The waitlist control group did not receive any training during the implementation of the intervention program in the experimental group. The control group received the skills-training program after completion of the postintervention measurement (T2), approximately eight weeks later than the experimental group.

### Instruments

#### Demographics

Characteristics of adolescents were collected at baseline (T1), including gender, age, number of siblings, country of birth, ethnic identity, educational level (and school year), living situation, and financial problems. We assessed *ethnic identity* by asking which identity the participants felt most connected to (I see myself as: “Dutch, Indonesian, Turkish, Moroccan, Surinamese, Antillean, other, or combination”) and distinguished three groups: Western (e.g., Dutch), mix Western-non-Western (e.g., Dutch-Turkish) or non-Western (e.g., Moroccan). *Educational level* of participants was assessed by the class they were in (e.g., practical education, preuniversity education). Three groups were distinguished: practical-prevocational education students (i.e., lowest educational level), prevocational/senior general education students, and senior general-preuniversity education students (i.e., highest educational level). *Living situation* was assessed by asking participants about their living situation (with answers options such as: “I live with both my parents, and my parents live together in one house”, “I live alone with my father/mother”, “I don’t live with either of my parents”, or “other”). Two groups were distinguished: living with both parents (adolescents who lived with both their parents in the same house) versus living in a non-nuclear family (adolescents who reported something else). *Financial problems* were assessed by asking participants about financial problems in their family.

#### Program Targets

*Skills to deal with performance anxiety* were assessed with the Dutch version of the Cognitive Emotional Regulation Questionnaire, short form (CERQ-short) (Garnefski & Kraaij, 2006). Research in adolescents and adults showed that the CERQ-short has adequate reliability and validity (Garnefski & Kraaij, 2006; Santos et al., 2021). Participants completed an 18-item self-report questionnaire measuring cognitive related coping, measured on a 5-point Likert scale ranging from 1 (*never*) to 5 (*always*). The questionnaire consists of nine subscales, divided into maladaptive and adaptive coping (Garnefski et al., 2001). The maladaptive coping scale consists of the subscales self-blame, other-blame, rumination, and catastrophizing (e.g., “I keep thinking about how terrible it is what I have experienced”). The adaptive coping scale consists of the subscales acceptance, refocus on planning, positive refocusing, positive reappraisal, and putting into perspective (e.g., “I think I can learn something from the situation”). Sum scores were computed for both subscales, with a higher score reflecting more maladaptive coping (α = 0.81 at T1 and α = 0.84 at T2; 8 items) and adaptive coping (α = 0.82 at T1 and α = 0.88 at T2; 10 items).

*Social skills* were assessed with the Scale for Interpersonal Behavior of Adolescents (SIG-A) (Arrindel et al., 1984; Bijstra & Oostra, 2000). Results show that the SIG-A is a reliable and valid instrument for indicating and evaluating social skills training for young adolescents (Bijstra & Oostra, 2000). Participants completed a questionnaire of 47 situations, evaluating how often they experienced these specific situations (i.e., frequency), based on a scale ranging from 1 (*never*) to 5 (*always*). The questionnaire consists of four subscales that refer to specific social situations: (1) display negative feelings (14 items, e.g., “If someone interrupts you, saying you find that annoying”), (2) express personal limitations (13 items, e.g., “Asking for an explanation about something that you did not understood”), (3) initiate assertiveness (9 items, e.g., “Starting a conversation with someone you have not met before”), and (4) display positive feelings (8 items, e.g., “Agreeing when someone makes a compliment about your appearance”). Sum scores were computed per subscale, with a higher score reflecting more display of negative feelings (α = 0.83 at T1 and α = 0.87 at T2), expression of personal limitations (α = 0.84 at T1 and α = 0.88 at T2), assertiveness (α = 0.84 at T1 and α = 0.87 at T2), and display of positive feelings (α = 0.81 at T1 and α = 0.85 at T2).

#### Direct Program Outcomes

*Performance anxiety* was assessed with two questionnaires, measuring fear of failure and test anxiety (i.e., anxiety in school testing situations). The Dutch short form of the Performance Failure Appraisal Inventory (PFAI short form) (Conroy et al., 2002) and the Dutch short version of the Spielberger Test Anxiety Inventory (TAI short form) (Spielberger, 1980; van der Ploeg, 1984) were used, respectively. The PFAI short form demonstrated adequate cross-validity and internal consistency in a college student sample (Conroy et al., 2002). The Dutch version of the TAI short form was found to have good psychometric properties, including sufficient stability, internal consistency and reliability in a student sample (van der Ploeg, 1984). The PFAI is a 5-item self-report questionnaire (e.g., “When I am failing, I am afraid that I might not have enough talent”), measured on a 5-point scale ranging from -2 (*do not believe at all*) to 2 (*believe 100% of the time*). A mean-item score was calculated. The TAI is a 20-item self-report questionnaire (e.g., “During tests I feel very tense”), measured on a 4-point scale ranging from 1 (*almost never*) to 4 (*almost always*). Sum scores were computed. Higher (positive) scores reflect more fear of failure (PFAI, α = 0.84 at T1 and α = 0.89 at T2) and test anxiety (α = 0.93 at T1 and α = 0.88 at T2).

*Social anxiety* was assessed with the social phobia scale of the Revised Child Anxiety and Depression Scale (RCADS) (Chorpita et al., 2000), Dutch version. The RCADS demonstrated sufficient convergent, discriminant, and factorial validity in a clinical sample of children and adolescents (Chorpita et al., 2005). Participants completed a 9-item self-report questionnaire (e.g., “I worry what other people think of me”), measured on a 4-point scale ranging from 0 (*never*) to 3 (*always*). Sum scores were computed, with a higher score reflecting more social anxiety (α = 0.89 at T1 and α = 0.90 at T2).

#### Mental Health Outcomes

*Stress levels* of participants were assessed with the Chronic Stress Questionnaire for Children and Adolescents (CSQ-CA) (de Bruin et al., 2018). The CSQ-CA showed good psychometric properties, including sufficient reliability and convergent validity, in a general population and clinical sample of adolescents (de Bruin et al., 2018). Participants completed a 17-item self-report questionnaire (e.g., “I often get upset about things that are not important”), measured on a 4-point scale ranging from 1 (*not true for me at all*) to 4 (*completely true for me*). Sum scores were computed, with a higher score reflecting more stress (α = 0.83 at T1 and T2).

*Internalizing and externalizing problems* were assessed with the Dutch version of the Youth Outcome Questionnaire (Y-OQ-30.1) (Dunn et al., 2005). Results demonstrated that the Y-OQ-30.1 has sufficient levels of internal consistency, test–retest and interrater reliability, investigated in community and outpatient adolescent samples (Dunn et al., 2005). The self-report questionnaire consists of 30 items, measuring psychological symptoms and social functioning on a 5-point Likert scale ranging from 0 (*never*) to 5 (*always*). It consists of six subscales:somatic complaints, social isolation, aggression, conduct problems, hyperactivity/distractibility, and depression/anxiety. Internalizing problems were assessed with the subscale depression/anxiety (six items, e.g., “I am sad or unhappy”). Externalizing problems were assessed with the subscales aggression and conduct problems (nine items, e.g., “I fight with adults”). Sum scores were computed for both subscales, with higher scores reflecting more internalizing problems (α = 0.83 at T1 and α = 0.85 at T2) and externalizing problems (α = 0.88 at T1 and α = 0.90 at T2).

*Self-esteem* was measured with the Dutch version of the Rosenberg Self-Esteem Scale (RSES) (Franck et al., 2008; Rosenberg, 1965). The Dutch RSES showed high internal consistency and congruent validity in adults (Franck et al., 2008) and other research showed sufficient psychometric properties for the RSES in adolescent samples (Bagley, 1997; Cong & Cheong, 2022). Participants completed a 10-item self-report questionnaire (e.g., “At times I think I am no good at all”), based on a 4-point scale ranging from 1 (*strongly agree*) to 4 (*strongly disagree*). Sum scores were computed, with a higher score reflecting more self-esteem (α = 0.87 at T1 and α = 0.86 at T2).

*Well-being* was measured with the WHO-Five Well-Being Index (WHO, 1998), Dutch version. Research demonstrated that the WHO-Five Well-Being Index has good psychometric properties, including concurrent validity and reliability, investigated in a clinical sample of adolescents (de Wit et al., 2007). Participants were asked to think about the past two weeks and report how they felt. The self-report questionnaire consists of five statements (e.g., “My daily life has been filled with things that interest me”), based on a 6-point scale ranging from 0 (*at no time*) to 5 (*all of the time*). The sum score was multiplied by four to create the final score (i.e., 0–100), with higher scores reflecting more positive well-being (α = 0.85 at T1 and α = 0.89 at T2).

#### Statistical Analyses

Statistical analyses were performed using SPSS version 25. Descriptive analyses were performed for all study variables. We followed an intention-to-treat approach, and included all participants in the analyses (including participants who did not start the intervention or attended only a few sessions) to reduce motivation as a potential confounding effect and to examine the effectiveness of the assigned program (Montori & Guyatt, 2001). To check whether the randomization was successful, differences between the experimental and control groups at T1 (e.g., demographics, mental health outcomes) were investigated by conducting independent t-tests and chi-square tests.

To test the effectiveness of both school-based skills-training programs, we considered multilevel analyses. However, because no nested structure was observed in the data (i.e., based on the different schools), we pursued with multivariate analyses of covariance (MANCOVA’s) for the three outcome domains (i.e., program targets, direct program outcomes, and mental health outcomes). The posttest outcome measures (i.e., at T2) were included as dependent variables and condition (i.e., experimental and control group) was included as a fixed factor. The pretest outcome measures (i.e., at T1) and potential differences between the experimental and control group at T1 were included as covariates in the analyses. MANCOVA’s that were (trend-)significant were followed by tests of between-subjects effects, to investigate whether there was a statistically significant difference between the groups in terms of each dependent variable. Cohen’s *d*’s were calculated using an online effect size calculator (Wilson, n.d.), based on standardized means and errors. A positive Cohen’s *d* reflects improvements favoring the experimental group compared to the control group. Small effect sizes were considered *d* = 0.20, moderate effect sizes *d* = 0.50 and large effect sizes *d* = 0.80 (Cohen, 1988).

As the COVID-19 pandemic affected this project, we performed some additional (sensitivity) analyses to determine its impact on the results of this study. First, we investigated whether cohort influenced the results. Almost half of the participants enrolled in the skills-training programs before the COVID-19 pandemic (Cohort 1; *N* = 144). The other participants enrolled during the pandemic (Cohort 2; *N* = 217). Of Cohort 2 participants, most (Cohort 2a; *N* = 181) registered before postponement of the programs (before March 2020), while some (Cohort 2b; *N* = 36) registered at a later opportunity, shortly before the postponed programs were to start. We performed a MANCOVA (as mentioned in the previous paragraph), with cohort as a covariate (i.e., cohort 1, 2a and 2b), adding the interaction of condition versus cohort (i.e., condition*cohort). Second, we repeated the MANCOVA’s in the subsamples of adolescents who attended more than four sessions (i.e., participants who attended more than 57% of the program; 46 participants (48.4%) in the performance anxiety program and 62 participants (71.3%) in the social skills program). Before the analyses, we examined differences at T1 between participants who attended more than four sessions and the control group. Materials, datasets, and analysis codes for this study are available from the corresponding author upon request.

## Results

### Differences at T1 Between Experimental and Control Group

There were no significant differences at T1 between the experimental and control group for both skills-training programs, except for gender in the performance anxiety program. There were more females in the experimental group (see Table 1). Hence, further analyses were performed with gender as a covariate (for the performance anxiety program only). Apart from this, the randomization was successful for both skills-training programs (see Tables 1, 2, 3).

**Table 2:** Descriptive statistics and MANCOVA results for the skills-training program targeting skills to deal with performance anxiety

Variables	Experimental group (*N* = 95)		Control group (*N* = 101)				
	Pretest (T1)	Posttest (T2)	Pretest (T1)	Posttest (T2)	Differences at T1	MANCOVA condition	
	*M* (*SD*)	*M* (*SD*)	*M* (*SD*)	*M* (*SD*)	*F* (*p*)	*F* (*p*)	Cohen’s* d*^a^
*Program target outcomes*						1.103 (.334)	
Maladaptive coping	17.63 (6.43)	17.79 (6.27)	18.62 (6.09)	19.45 (6.65)	− 1.109 (.269)		0.19
Adaptive coping	27.74 (8.11)	27.24 (8.60)	28.28 (7.70)	28.82 (8.64)	− 0.478 (.633)		− 0.20
*Direct program outcomes*						2.733 (.068)^*b*^	
Test anxiety	40.93 (11.7)	38.85 (10.60)	42.37 (13.04)	42.26 (12.69)	− 0.810 (.419)		0.32
Fear of failure	− 0.80 (0.94)	− 0.86 (0.97)	− 0.88 (0.89)	− 0.75 (0.92)	0.620 (.536)		0.28
*Mental health outcomes*^*c*^						0.843 (.521)	
Stress	40.80 (7.89)	40.14 (8.38)	40.04 (8.41)	41.02 (7.84)	0.650 (.516)		0.29
Internalizing problems	6.01 (4.74)	6.07 (4.80)	6.63 (5.06)	7.05 (5.36)	− 0.881 (.379)		0.17
Externalizing problems	4.27 (4.18)	5.21 (5.92)	5.59 (5.69)	6.63 (6.53)	− 1.846 (.067)		0.05
Self-esteem	29.35 (5.17)	28.83 (4.96)	29.16 (6.06)	27.97 (6.18)	0.233 (.816)		0.19
Well-being	56.80 (22.34)	57.39 (25.26)	57.44 (23.66)	55.68 (24.40)	− 0.194 (.846)		0.16

Analyses were performed with gender as covariate

^a^Cohen’s *d’s* were calculated based on standardized means and errors

^b^Follow-up tests of between-subject effects were performed

^c^*N* = 100 for the control group, because one participant did not complete the questionnaires

**Table 3:** Descriptive statistics and MANCOVA results for the skills-training program targeting social skills

Variables	Experimental group (*N* = 86)		Control group (*N* = 79)				
	Pretest (T1)	Posttest (T2)	Pretest (T1)	Posttest (T2)	Differences at T1	MANCOVA condition	
	*M* (*SD*)	*M* (*SD*)	*M* (*SD*)	*M* (*SD*)	*F* (*p*)	*F* (*p*)	Cohen’s* d*^a^
*Program target outcomes*						0.221 (.927)	
Display of negative feelings	40.45 (9.02)	41.37 (10.29)	41.10 (9.88)	41.95 (12.46)	− 0.440 (.660)		− 0.01
Express personal limitations	42.65 (8.41)	41.65 (10.28)	42.58 (9.13)	42.25 (11.03)	0.050 (.960)		− 0.08
Initiate assertiveness	27.21 (6.86)	26.29 (7.48)	26.35 (7.67)	26.29 (9.01)	0.756 (.451)		− 0.13
Display of positive feelings	24.78 (6.58)	23.87 (7.24)	24.81 (6.79)	24.08 (7.77)	− 0.030 (.976)		− 0.03
*Direct program outcomes*						1.230 (.269)	
Social anxiety	7.97 (6.19)	7.52 (5.96)	8.42 (6.81)	8.53 (6.53)	− 0.447 (.655)		0.17
*Mental health outcomes*^*b*^						0.340 (.888)	
Stress	39.05 (7.73)	38.78 (7.50)	40.10 (8.64)	40.04 (8.63)	− 0.822 (.412)		0.09
Internalizing problems	5.55 (4.83)	6.05 (5.50)	6.21 (5.20)	6.74 (5.49)	− 0.831 (.407)		0.06
Externalizing problems	5.62 (5.94)	6.01 (7.46)	7.43 (8.01)	7.00 (6.90)	− 1.638 (.103)		− 0.02
Self-esteem	28.66 (6.16)	28.66 (5.94)	28.52 (6.38)	28.21 (6.28)	0.141 (.888)		0.09
Well-being	63.39 (23.69)	61.65 (24.41)	62.23 (21.15)	57.30 (29.02)	0.326 (.745)		0.16

^a^Cohen’s *d’s* were calculated based on standardized means and errors

^b^*N* = 85 for the experimental group and *N* = 77 for the control group, because two participants did not complete the questionnaires

### Program Target Outcomes

Tables 2 and 3 present the MANCOVA results of the performance anxiety program and social skills program, respectively. Results demonstrated that for the performance anxiety program, there were no significant effects for coping skills (i.e., maladaptive and adaptive coping). The social skills program yielded no significant effects for social skills (i.e., display of negative feelings, express personal limitations, initiate assertiveness, and display of positive feelings).

### Direct Program Outcomes

Results demonstrated that for the performance anxiety program only, there was a trend-significant effect of condition (*F* (2, 190) = 2.733, *p* = 0.068, η_p_^2^ = 0.028; Table 2). Between-subject results demonstrated a small but significant positive effect for test anxiety (*F* (1, 191) = 5.199, *p* = 0.024, *d* = 0.32), indicating that participants in the intervention group showed a significant reduction in test anxiety compared to participants in the control group. For the social skills program, there was no significant effect on social anxiety (Table 3).

### Mental Health Outcomes

For both the performance anxiety program and the social skills program, no significant effects were found for mental health outcomes (i.e., stress, internalizing and externalizing problems, self-esteem, and well-being) (Tables 2, 3).

### Sensitivity Analyses

For both skills-training programs, there were no significant condition*cohort effects for any of the outcomes, suggesting that program effectiveness was not affected by COVID-19-related postponement of the programs. Next, for both programs, the MANCOVA’s were repeated with the subgroup of the experimental group consisting of participants who attended at least four program sessions compared to the control group. Apart from higher levels of fear of failure (*t* = 2.750, *p* = 0.007) and stress (*t* = 2.210, *p* = 0.029) in the experimental subgroup relative to the control group of the performance anxiety program, these experimental subgroups did not differ from the control groups on any of the T1 assessments (i.e., for both programs). The MANCOVA’s yielded similar results for the social skills program (i.e., no significant effects), whereas the aforementioned significant results for the performance anxiety program were slightly stronger. That is, a significant effect was found for the performance anxiety program (*F* (2, 142) = 3.824, *p* = 0.024, η_p_^2^ = 0.051) and between-subject results demonstrated that the program effectively reduced test anxiety (*F* (1, 143) = 5.777, *p* = 0.018, *d* = 0.41) and fear of failure (*F* (1, 143) = 4.710, *p* = 0.032, *d* = 0.38). This indicates that participants who attended more than four sessions of the performance anxiety program showed significant reductions in test anxiety and fear of failure compared to participants in the control group. All other results remained unchanged.

## Discussion

Given that high levels of stress during adolescence are associated with negative mental health consequences and adolescents are exposed to various stressors related to school and social situations (Núñez-Regueiro & Núñez-Regueiro, 2021), interventions at school focusing on stress reduction (i.e., school or social stress) may be an appropriate way to address adolescents’ psychological needs. Therefore, the current study aimed to examine the effectiveness of two targeted school-based skills-training programs, addressing either skills to deal with performance anxiety or social skills. Results demonstrated that the performance anxiety program had a small effect on reducing adolescents’ test anxiety. Furthermore, when adolescents attended more than four sessions (i.e., more than 57% program attendance), the program had a small effect on reducing levels of test anxiety and fear of failure. The performance anxiety program did not improve adolescents’ coping skills (i.e., adaptive and maladaptive coping) nor mental health outcomes (i.e., stress, internalizing and externalizing problems, well-being, and self-esteem). The social skills program was not effective in improving any of the outcomes, including social skills (i.e., display of negative feelings, express personal limitations, initiate assertiveness, and display of positive feelings), social anxiety, and mental health.

The finding that the performance anxiety program had a small effect on reducing test anxiety is in line with previous research in adolescents (O’Driscoll & McAleese, 2021; Putwain & von der Embse, 2021; Soares & Woods, 2020; von der Embse et al., 2013), highlighting that such programs have the potential to support adolescents who express a need for support in this area. Furthermore, our results demonstrated positive effects on test anxiety and fear of failure for adolescents who attended more than four sessions. As attending more than half of the sessions increased program effectiveness, additional efforts should be made to motivate students to attend and engage in such programs. A previous study demonstrated that motivational interviewing (e.g., explicit attention to participants hopes, experience with previous mental health treatment, external difficulties, and internal barriers) before the start of cognitive behavioral therapy enhanced treatment engagement in adolescents (Dean et al., 2016). More motivation may increase program attendance and engagement, which is likely to enhance program effectiveness, and should thus be encouraged. Although the effects were small, the performance anxiety program consisted of only seven sessions, demonstrating that even a relatively short performance anxiety program can yield positive results in reducing adolescents’ performance anxiety. Yet, previous intervention studies showed moderate to large effects for reductions in test anxiety, for interventions with similar intensity (O’Driscoll & McAleese, 2021; Weems et al., 2014). The stronger effects might be related to a different screening method (i.e., inclusion based on screening; Weems et al., 2014) or a different intervention (i.e., compassionate mind training, with a focus on a prosocial approach; O’Driscoll & McAleese, 2021). Overall, it appears that school-based intervention programs addressing skills to deal with performance anxiety are beneficial for adolescents with psychological needs (i.e., a self-selected sample). This is promising, as school-based stress among adolescents increased over the last two decades (Boer et al., 2021; Cosma et al., 2020, 2021; Stevens et al., 2018) and high levels of test anxiety are associated with negative consequences, such as lower educational performance and self-esteem (von der Embse et al., 2017). Hence, governments and schools should be aware that school-based performance anxiety programs have some potential to reduce adolescents’ test anxiety and fear of failure, and should consider offering such programs to students that suffer from school-related stress.

Alternatively, given the higher levels of stress and fear of failure for adolescents who attended more than four sessions of the performance anxiety program relative to participants in the control group, the somewhat stronger effects in the sensitivity analysis may point towards more effectiveness for adolescents with more or greater needs, who may also be more motivated. A recent study also indicated that higher levels of baseline problems were associated with greater treatment effectiveness in adolescents (Stjerneklar et al., 2019). This suggests that adolescents with higher initial problems may have more to gain from intervention programs, which advocates the use of screening methods (e.g., self-selection) at recruitment. Further research is necessary to draw clear conclusions about the effects of initial problem severity on program effectiveness.

Interestingly, we found that the performance anxiety program improved performance anxiety without improving adolescents’ coping skills. This suggests that improving coping skills was not the working mechanism by which adolescents’ performance anxiety was reduced. It could be that other mechanisms were involved in reducing performance anxiety, such as increased understanding (from psycho-educative elements) or ability to relax (from relaxation techniques). Previous research, for example, demonstrated that relaxation exercises reduced test anxiety in children and adolescents (Gregor, 2005; Larson et al., 2010). Another explanation for not finding improvements in coping skills could be that participants needed more time to internalize the newly learned coping skills, and only after some additional time would report significant changes (i.e., after experiencing some challenging situations). In that case, immediate postintervention effects would be smaller or less probable than effects in the long-term (i.e., at follow-up). Therefore, future studies should also include follow-up measurements, to identify sleeper effects (i.e., improved longer term outcomes). Finally, it is also possible that our questionnaire did not fully capture skills to deal with performance anxiety, as the questionnaire was related to coping skills in general rather than specifically related to performance anxiety. Future effectiveness research should include a questionnaire that is more specific to coping with performance anxiety. For instance, questions could be developed that take into account the context of performance or test anxiety situations (e.g., dealing with exam failure, giving a presentation).

With regard to mental health outcomes, the current study showed that the performance anxiety program did not improve adolescents’ mental health (i.e., stress, internalizing and externalizing problems, self-esteem, and well-being), which is inconsistent with most previous literature (Bradley et al., 2010; O’Driscoll & McAleese, 2021; Yahav & Cohen, 2008). However, one study demonstrated that effects on secondary mental health symptoms were seen later at follow-up, as a function of change in test anxiety (Weems et al., 2014). Hence, it is possible that we did not find positive effects on (secondary) mental health outcomes because we did not measure these outcomes at a later stage (i.e., at follow-up). Moreover, since mental health problems were secondary or distal program outcomes, smaller effects could be expected immediately postintervention. Further, it could be that more sessions are necessary to observe significant mental health changes among adolescents. Indeed, previous test anxiety interventions with demonstrated positive mental health changes had more or longer sessions (Bradley et al., 2010; Yahav & Cohen, 2008). Nevertheless, although we did not observe significant positive effects on coping skills or mental health outcomes, there were no detrimental program effects. This suggests that the targeted school-based performance anxiety program is promising as a preventive intervention for adolescents to reduce performance anxiety immediately after completion of the program.

The current study demonstrated that the targeted school-based social skills program was not effective in improving social skills, nor reducing social anxiety or improving mental health outcomes. This seems to contradict previous results, that observed positive effects for school-based social skills programs (Durlak et al., 2011; Gaspar et al., 2018; Schonert-Reichl & Lawlor, 2010). However, these programs were mostly more intensive interventions, with respectively 10 sessions (Schonert-Reichl & Lawlor, 2010), 22 sessions (Gaspar et al., 2018), and a mean number of 40.8 sessions (Durlak et al., 2011). It could therefore be that we did not observe significant program effects because the intervention was too short or did not contain enough exercises. Indeed, a recent meta-analysis demonstrated that an optimal social skills intervention for nonclinical children and adolescents should contain three to six psychoeducation components (e.g., transferring knowledge) and 11–20 skill-building exercises (e.g., teamwork exercises; de Mooij et al., 2020), which is more than the number of exercises in the current social skills program. Future research should take this into account and develop school-based interventions with an optimal number of sessions and components.

Alternative explanations for the absence of effectiveness of the social skills program are the heterogeneous group of participants with relatively mild social deficits, the broad scope of the program, and the limited transfer of skills to daily life. First, as noticed by some of the trainers, it is possible that the participants in the training groups were too heterogeneous, regarding problems before the start of the program (e.g., social anxiety, impulsive or aggressive behavior) and/or educational levels (e.g., practical education, preuniversity education), which might have disrupted the dynamics of the group and complicated the teaching process. Second, it is possible that the target group experienced relatively mild social deficits, which makes it more challenging to demonstrate program effects. Third, the social skills program consisted of teaching different social skills (e.g., assertiveness, emotion regulation, self-esteem). Possibly, this broad rather than in-depth focus did not allow for significant improvements. Lastly, in order to generate positive changes, it may be necessary to practice and apply the learned social skills in everyday life, as previous research demonstrated that prevention programs with homework assignments yielded larger effects than programs without (Stice et al., 2009). Although adolescents were encouraged to practice their skills in daily life, there were no official homework assignments in the current study, which may have impeded transfer of skills in daily life. Given that few studies examined the effectiveness of targeted school-based social skills programs, further intervention studies are needed to draw conclusions about the effectiveness of such programs and effective elements in particular.

### Strengths and Limitations

The current study has some limitations. First, as we used a waitlist control group, long-term follow-up measurements were not possible. This is a limitation, as a previous meta-analysis investigating the effectiveness of school-based stress-reduction interventions demonstrated larger effects for follow-up compared to postintervention assessments (van Loon et al., 2020). It could be that improvements in (mental health) outcomes only manifest at a later stage in the adolescents’ life (i.e., sleeper effect), not immediately after the intervention. Moreover, it is possible that the absence of follow-up measurements hindered observing long-term positive (mental health) outcome effects, as previous research showed that test anxiety interventions reduced mental health problems not immediately after the intervention but at a later stage (at follow-up; Weems et al., 2014). Future intervention research should therefore also include follow-up assessments, to examine long-term effects.

Second, the usual challenges of program implementation (e.g., participant recruitment and attainment, collaboration with stakeholders, practical issues) were aggravated by the COVID-19 pandemic. As the schools had to close (suddenly), the skills-training programs at six schools were postponed and restarted, and most participants had to complete the T1 assessments again. Consequently, a subgroup of participants had to fill in the questionnaires more often than (the original) two times to control for time effects and some had to restart the intervention program at least six months later than planned. Postponement of the programs may have resulted in a lower program attendance (particularly for the performance anxiety program), for instance because adolescents felt they did not need the extra support anymore (e.g., because they were in a higher grade or were not experiencing problems), they did not want to miss any classes (i.e., the programs were mostly scheduled during school hours), or because of lack of motivation in general. In sum, implementation challenges due to the COVID-19 pandemic (e.g., multiple assessments, low program attendance), might have influenced program effectiveness. Future research should include focus groups with students, as this may provide valuable information about experienced barriers to program attendance that should be considered in the development and implementation of school-based intervention programs. Nevertheless, program enrollment before or after the COVID-19 pandemic did not yield different program effects, suggesting that the COVID-19 pandemic-induced challenges had little impact on program effectiveness.

Additionally, despite the COVID-19 pandemic and difficulties of recruiting hard-to-reach participants, such as ethnic minority groups and groups of lower socioeconomic status (Bonevski et al., 2014), we recruited a large, diverse sample of adolescents aged between 12 and 17 years. Adolescents were recruited from nine secondary schools located in one of the four largest cities in the Netherlands, and consisted of adolescents from various cultural backgrounds (about half had a non-Western ethnic identity) and educational levels (ranged from practical to preuniversity education). Moreover, our sample was comparable to Dutch adolescents (10–15 years) regarding minority background (CBS, 2021a) and educational level (CBS, 2021b). As such, our findings are likely representative for a broad group of (Dutch) adolescents.

## Conclusion

In conclusion, the current study examined two targeted school-based skills-training programs by addressing school or social stress, yielding positive effects for the performance anxiety program, but no improvements for the social skills program. The performance anxiety program was effective in reducing adolescents’ performance anxiety, indicating that a relatively short performance anxiety program can be beneficial for adolescents. The social skills program was not effective in improving social skills, social anxiety, and mental health, probably because the program was too broad and/or not sufficiently intensive. Recent meta-analyses showed that social skills interventions for nonclinical children and adolescents should contain an optimal number of (psychoeducation and skill-building) components and sessions (de Mooij et al., 2020) and that some components of school-based interventions show stronger or weaker program effects (Mertens et al., 2020). Hence, new intervention programs should include components that are related to stronger effects on relevant outcomes and match the target group, and existing programs may be improved by adding or removing components, or changing the order of (effective) components. This study, in combination with recent published meta-analyses (de Mooij et al., 2020; Mertens et al., 2020), could serve as a baseline to improve and optimize the effectiveness of school-based intervention programs. For instance, longer sessions or more exercises seem necessary for social skills training programs, with an optimal number of three to six psychoeducational and 11–20 skill-building exercises (de Mooij et al., 2020). Furthermore, teaching emotion regulation and assertiveness are related to weaker program effects, while teaching self-awareness and problem-solving are related to stronger program effects (Mertens et al., 2020). Future projects should take this into account when developing, implementing and examining school-based skills-training programs for adolescents. In both programs, participation was based on self-selection, demonstrating that adolescents (with psychological needs) were willing to sign up for a low threshold skills-training program at school. This is important, as the majority of adolescents with mental health problems do not receive treatment (Merikangas et al., 2011). Given the few positive results for the performance anxiety program, and the fact that school mental health services are associated with lower stigma (Stephan et al., 2007), school-based performance anxiety programs have some potential to be beneficial for adolescents during the first years of secondary school. Nevertheless, follow-up research is needed to establish long-term effects, as well as the effective ingredients of such programs.

## Supplementary Information

Below is the link to the electronic supplementary material.

Supplementary file1 (PDF 40 KB)

## Data Availability

The datasets generated and analyzed during the current study are not publicly available but are available from the corresponding author on request.

## Footnotes

1 Participants were excluded because whole classes were signed up (*N* = 56), participants did not receive the psychoeducation lessons (*N* = 3), participants signed up twice (second participation was excluded; *N* = 2), participants received a different intervention (*N* = 4), or because participants switched condition (experimental to control group; *N* = 2 and control to experimental group, *N* = 1). Three participants switched training group, but were not excluded (because they remained in the same condition).
