# Supplementary material for: The Effectiveness of School-Based Skills-Training Programs Reducing Performance or Social Anxiety: Two Randomized Controlled Trials
Source: Child Youth Care Forum. 2023 Feb 4:1–25. Online ahead of print. doi: 10.1007/s10566-023-09736-x (PMC9899115; doi:10.1007/s10566-023-09736-x)
Supplement: Supplementary file 1 — Supplementary file1 (PDF 40 KB) [file 10566_2023_9736_MOESM1_ESM.pdf]

The Effectiveness of School-Based Skills-Training Programs Reducing Performance or Social Anxiety: Two Randomized Controlled Trials.

Authors: Amanda W. G. van Loon [a], Hanneke E. Creemers [b], Simone Vogelaar [c], Anne C. Miers [c], Nadira Saab [d], P. Michiel Westenberg [c] & Jessica J. Asscher [a]

Corresponding author: Amanda W. G. van Loon, Child and Adolescent Studies (CAS), Utrecht University, Heidelberglaan 1, 3584 CS Utrecht, the Netherlands; Email: a.w.g.vanloon@uu.nl; Telephone number: +3130-2534801.

Journal: Child & Youth Care Forum

Supplementary Table 1

	Before the COVID-19 pandemic (*N* = 144)			After the start of the COVID-19 pandemic (*N* = 217)			
	School year 2018/2019	School year 2019/2020	Time T1	School year 2020/2021 (restarted)	School year 2020/2021 (postponed)	Time T1	Registered in September 2020 (*N* = 36, 16.6%)
	N (% of total)	*N* (% of total)		*N* (% of total)	*N* (% of total)		*N* (% of school)
School 1	50 (34.7)		February 2019	40 (18.4)		September 2020	4 (10.0)
School 2	3 (2.1)		February / March 2019	6 (2.8)		September 2020	0 (0.0)
School 3	36 (25.0)		May 2019	Dropped out			
School 4		20 (13.9)	December 2019				
School 5		35 (24.3)	January 2020				
School 6				111 (51.2)		October 2020	2 (1.8)
School 7				19 (8.8)		October 2020	1 (5.3)
School 8					10 (4.6)	September 2020	7 (70.0)
School 9					31 (14.3)	October 2020	22 (71.0)

Overview of Participating Schools, Final Number of Participants, and Data Collection Cohorts
